# Supplementary material for: Research on the impact of equity incentive model on enterprise performance: A mediating effect analysis based on executive entrepreneurship
Source: PLoS One. 2024 Apr 5;19(4):e0300873. doi: 10.1371/journal.pone.0300873 (PMC10997110; doi:10.1371/journal.pone.0300873)
Supplement: Attachment — Submitted filename: renamed_7346e.docx [file pone.0300873.s001.docx]

Dear Editors and Reviewers:

Thank you for your letter and for the reviewers’ comments concerning our manuscript entitled “Research on the Impact of Equity Incentive Model on Enterprise Performance: A Mediating Effect Analysis Based on Executive Entrepreneurship” (Manuscript Number: PONE-D-23-16055). We sincerely thank the editor and all reviewers for their valuable feedback that we have used to improve the quality of our manuscript. These comments are all valuable and helpful for improving our article. According to your nice suggestions, we have made extensive corrections to our previous manuscript. The detailed corrections are listed below. The revised parts of the article have been marked up using the Word Microsoft Track Changes tool. For the convenience of reviewers, we have uploaded two versions: manuscript-**Revised Manuscript with Track Changes (Word Microsoft Track Changes)** and manuscript- **Manuscript**.

**Answers to Review Report (Manuscript Number:** **PONE-D-23-16055):**

**1.** **Reviewer #1**

**Q1:** Abstract. What is the limitation of the existing studies? The authors need to explore the originality and contribution of the study in this section.
**Response:**
Thanks for your suggestion. Additional information about the limitations, originality, and contributions of the study has been provided in the abstract.

**Limitations:**
**See the manuscript – Manuscript: lines 29-32.**
or **the manuscript – Revised Manuscript with Track Changes (Word Microsoft Track Changes): lines 52-55. it is marked in yellow.**

**Originality:
See the manuscript – Manuscript: lines 10-16.**
or **the manuscript – Revised Manuscript with Track Changes (Word Microsoft Track Changes): lines 33-39. it is marked in yellow.**

**Contribution:
See the manuscript – Manuscript: lines 32-34.**
or **the manuscript – Revised Manuscript with Track Changes (Word Microsoft Track Changes): lines 55-57. it is marked in yellow.**

**Q2:** The introduction section lacks research gaps presentation. What are research gaps in existing literature? The authors should explicitly state in this section and then draw your contributions smoothly.
**Response:**
We believe that your suggestions are very useful to us. We have described the gaps between this study and the existing literature in the introduction section.

**See the manuscript – Manuscript: lines 78-85.**
or **the manuscript – Revised Manuscript with Track Changes (Word Microsoft Track Changes): lines 181-188. it is marked in yellow.**

**Q3:** The authors should provide a paragraph at the end of the Introduction section to present the article organization.
**Response:**
Thanks for the suggestion. We have added an introduction to the organization of the article at the end of the introduction.

**See the manuscript – Manuscript: lines 98-110.**
or **the manuscript – Revised Manuscript with Track Changes (Word Microsoft Track Changes): lines 201-213. it is marked in yellow.**

**Q4:** The literature section is weak; How the existing studies related to your methods? These issues should be stated clearly in this section.
**Response:**
We think your comments are very helpful. Based on the comments you made, we think it is very necessary to add the Literature Review section, so we added the Literature Review section. And we have summarized the existing research methods for this issue and explained the research methodology of this paper. For details, see 2. Literature Review.

**See the manuscript – Manuscript: lines 112-195.**
or **the manuscript – Revised Manuscript with Track Changes (Word Microsoft Track Changes): lines 215-298. Due to the excessive length of the section, it is not marked in yellow.**

**Q5:** The data source should be stated more clearly, and how the authors pre-process the null data?
**Response:**
Thanks for the heads up, We have added the treatment of null data in the data sources section.

**See the manuscript – Manuscript: lines 395-402.**
or **the manuscript – Revised Manuscript with Track Changes (Word Microsoft Track Changes): lines 504-513. it is marked in yellow.**

**Q6:** The discussion section is missed; the authors should focus on your results, or the recommendations should be drawn from your results directly.
**Response:**
Thanks to your suggestion, we have revised both the title and the content of the last section of the article, which contains results, discussion and conclusion. The section on results and recommendations focuses on **6.2 Conclusion**, which first summarizes the results of the paper and then makes relevant recommendations based on the results.

**Results section:**
**See the manuscript – Manuscript: lines 729-734.**
or **the manuscript – Revised Manuscript with Track Changes (Word Microsoft Track Changes): lines 853-858. it is marked in yellow.**

**Recommendation section:**
**See the manuscript – Manuscript: lines 739-742.**
or **the manuscript – Revised Manuscript with Track Changes (Word Microsoft Track Changes): lines 863-866. it is marked in yellow.**

**Q7:** The limitations or future research directions should be presented in a new subsection.
**Response:**
Thanks to your suggestions, we have added comparisons and discussions with the existing literature and have proposed future research directions for this paper in a separate subsection.

**See the manuscript – Manuscript: lines 743-748.**
or **the manuscript – Revised Manuscript with Track Changes (Word Microsoft Track Changes): lines 867-872. it is marked in yellow.**

**Q8:** Please updated some newest references.
**Response:**
We have updated the references based on the suggestions you have made. See References 11,16,20,23,29,30,42,46,52,55,64,65 and 66 for details.

**2.Reviewer #2:**

**Q1:** The authors should check the grammar and tense throughout the whole manuscript.
**Response:**
Thanks to your valuable comments, we have checked the manuscript for grammar and tenses and have touched up key parts of the paper. Details are given in **Word Microsoft Track Changes**.

**Q2:** The authors should check the language throughout the whole manuscript.
**Response:**
Thank you for your input for us, we have checked the language of the manuscript and made changes to some of the statements. See **Word Microsoft Track Changes** for details.

**Q3:** The authors should check the keywords and keep the order of the keywords based on the capital letter “A-Z” and 4-5 preferred.
**Response:**
Thanks for the suggestion. We kept the keywords to 4 or 5 and sorted them in A-Z order.
**See the manuscript – Manuscript: lines 35-36.**
or **the manuscript – Revised Manuscript with Track Changes (Word Microsoft Track Changes): lines 58-59, it is marked in yellow.**

**Q4:** In the introduction, the authors should pay more attention to the recent and important literature. The following is upon the topic “Equity Incentive, Enterprise Performance, Executive Entrepreneurship” that the authors can cite, I suggest that
Does venture capital help to promote open innovation practice? Evidence from China. <https://doi.org/10.1108/EJIM-03-2021-0161>
Effect of Founder Control on Equity Financing and Corporate Performance-Based on Moderation of Radical Strategy. <https://doi.org/10.1177/21582440221085013>
Military Experience and Individual Entrepreneurship—Imprinting Theory Perspective: Empirical Evidence From China. https://doi.org/10.1177/21582440231159866
**Response:**
Thanks to your valuable comments, we have cited the three references you recommended in the manuscript and reworked the introductory section. The cited references can be found in references 42,43,55.

**See the manuscript – Manuscript: lines 341-342,383-384 and 739-742.**
or **the manuscript – Revised Manuscript with Track Changes (Word Microsoft Track Changes): lines 445-446,492-493 and 863-866. it is marked in yellow.**

**Q5:** The authors should add value and process of the study in the section.
**Response:**
Based on the revisions you have suggested, we have included the research value and process in the introduction section.

**Value：**
**See the manuscript – Manuscript: lines 86-97.**
or **the manuscript – Revised Manuscript with Track Changes (Word Microsoft Track Changes): 165-180.**

**Process：
See the manuscript – Manuscript: lines 98-110.**
or **the manuscript – Revised Manuscript with Track Changes (Word Microsoft Track Changes): lines 201-213. it is marked in yellow.**

**Q6:** The authors should add more details in the methodology section and how to collect data.
**Response:**
Following your suggestions, we have added to the text how to collect data and the processing of vacancy data.
**See the manuscript – Manuscript: lines 397-402.**
or **the manuscript – Revised Manuscript with Track Changes (Word Microsoft Track Changes): lines 508-513, it is marked in yellow.**

**Q7:** The authors can change the expression of fig. 1.
**Response:**
Thank you for your revision, we have changed the presentation of Figure 1 in the text.

**Q8:** The authors add the meaning of *, **, ** in table 8 and table 9, table 10, table 11 and table 12 and table 13. **Response:**
Thanks to your suggestion, we have added *, **, ** to Tables 8 and 9, 10, 11 and 12 and 13.

**Q9:** The authors should add more to the “Discussion section”, meanwhile, comparing the result with previous ones. The authors should write the discussion section, however, it is missing.

**Response:**
In response to your suggestion, we have added the “Discussion section” to Part 6 of the article and compared the results of this article with previous results.
**See the manuscript – Manuscript: 647-727.**
or **the manuscript – Revised Manuscript with Track Changes (Word Microsoft Track Changes): 771-851. Due to the excessive length of the section, it is not marked in yellow.**

**Q10:** The authors should add conclusion section, however, it is also missing.
**Response:**
Thank you for your advice, which is reflected in the conclusion section of the article.
**See the manuscript – Manuscript: 729-734.**
or **the manuscript – Revised Manuscript with Track Changes (Word Microsoft Track Changes): 853-858.**

**Q11:** The authors should check the important and recent ones.
**Response:**
We have updated the references based on the suggestions you have made. See References 11,16,20,23,29,30,42,46,52,55,64,65 and 66 for details.

**Q12:** The authors should check all the references’ formats based on Plos One’s style. Besides, some lack pages. Besides, the authors should pay more attention to the text-in ciatations through the whole manuscript again and check strictly one by one.
**Response:**
Thanks to your reminder, we have rechecked the formatting of all references according to Plos One style and have checked the citation of this article.

Thank you for your precious comments and advice. Those comments are all valuable and very helpful for revising and improving our paper. We have revised the manuscript accordingly, and our point-by-point responses are presented above.

Yours sincerely,

Jinrong Ma

Address: Liaoning University of Science and Technology, Anshan City, China

Tel: +86-15638976977

E-mail: 15638976977@163.com
